# Retrospective and prospective measures of post-traumatic growth reflect different processes: longitudinal evidence of greater decline than growth following a hematopoietic stem-cell transplantation

**DOI:** 10.1186/s12888-020-03007-y

**Published:** 2021-01-11

**Authors:** Maya Corman, Marie-Thérèse Rubio, Aurélie Cabrespine, Isabelle Brindel, Jacques-Olivier Bay, Régis Peffault De La Tour, Michaël Dambrun

**Affiliations:** 1 Université Clermont Auvergne (UCA), LAPSCO CNRS, 34 avenue Carnot, 63037 Clermont-Ferrand, France; 2 Service d’hématologie, CHRU Nancy- Hôpitaux de Brabois, 54511 Vandoeuvre-les-Nancy, France; 3 CHU de Clermont-Ferrand, site Estaing, service de thérapie cellulaire et d’hématologie clinique adulte, 63000 Clermont-Ferrand, France; 4 Hôpital Saint-Louis, service d’hématologie, greffe de moelle, 75010 Paris, France

**Keywords:** Hematopoietic stem cell transplantation, Post-traumatic growth, Mental health, Psychological disposition, Post-traumatic stress disorder

## Abstract

**Background:**

This prospective longitudinal study examined and compared two measures (prospective and retrospective ones) of post-traumatic growth (PTG) following Hematopoietic Stem-Cell Transplantation (HSCT) and their respective relationships with mental health and psychological disposition. We also tested the hypothesis that unwillingness to be in contact with distressing thoughts and feelings—i.e. experiential avoidance—would moderate the relationship between Post-Traumatic Stress Disorder (PTSD) and growth.

**Methods:**

This study was carried out with 187 patients. Patients completed the Post-Traumatic Growth Inventory (PTGI) 5 months after HSCT and scales tapping into the five domains of PTGI during hospitalisation and 5 months after HSCT. Mental health and psychological disposition were also assessed prior to hospitalisation. A PTSD scale was administered at the five-month follow-up.

**Results:**

Prospective and retrospective measures of PTG were weakly correlated. Bayesian pre/post-HSCT comparisons in the prospective measure of PTG revealed substantial to very strong decline in four of the five dimensions assessed. Overall, RCI indicated a reliable increase for 5.6% of patients and a reliable decrease for 40.8% of patients. Confirming that retrospective and prospective measures of PTG reflect different processes, they were not related to the same mental health and psychological disposition variables. Moreover, the hypothesis that acquiring positive outcomes from a potentially traumatic experience, such as HSCT, requires direct confrontation with the source of distress was supported in the case of the retrospective measure of growth but not in the case of the prospective measure growth.

**Conclusions:**

Retrospective measures such as the PTGI do not appear to assess actual pre- to post-HSCT change. HSCT seems more linked to psychological decline than to growth.

## Background

Hematological malignancies and the side effects of treatments are particularly distressful and disturbing for the mental health and quality of life of patients [1, 2]. Hematopoietic Stem Cell Transplantation (HSCT) is associated with fatigue, pain, anxiety, depression, and Post-Traumatic Stress Disorder (PTSD) [3–5]. However, several studies suggest that cancer and its treatment may be accompanied by a process of psychological recovery and growth. Some theorists even propose that growth requires the presence—and a direct confrontation with the source—of distress [6–8]. However, problematically, this area of research is subject to significant criticism and controversy, raising important issues not only about the nature of post-traumatic psychological growth following cancer and its treatment, but also about the processes involved [9–13].

Post-traumatic growth refers to a positive psychological change following a traumatic life event [8]. The Post-Traumatic Growth Inventory (PTGI) is the most commonly used instrument to assess growth following an adverse event [14]. However, it does not assess actual change but self-perceived growth. Numerous studies have highlighted the limitations of retrospective self-perceived measures such as the PTGI, which were recently summarised by Infurna and Jayawickreme [12] as follows: ‘PTGI may reflect meaningful personality change to some degree but also maladaptive reality distortions, selective appraisals, coping strategies, personality characteristics, ways of explaining emotion levels, reflections of people’s implicit theories of change, and beliefs that their past selves were worse than they actually were’ (pp. 3–4). The Janus model proposed two components of PTGI: a constructive component and an illusory one [15]. The illusory nature of PTGI was empirically supported by the study by Frazier et al. [11], who concluded that this type of instrument does not appear to measure actual change. Furthermore, when comparing scores for actual change between before and after trauma, Frazier et al. did not find more evidence for reliable psychological growth than decline, raising issues about the characteristics of genuine post-traumatic growth and the best measure to describe it. This lack of robust empirical evidence, accompanied by significant methodological limitations, led Infurna and Jayawickreme [12] to recommend (a) caution when interpreting studies using retrospective self-perceived measures, and (b) the use of prospective longitudinal designs that allow a more reliable assessment of the change between before and after the adverse event. However, it is important to point out that pre/post-test designs also present some limits regarding the principles of response shift theory [16]**,** which emphasises that changes in self-reported measures that occur over time can reflect a recalibration of internal standards of self-perception. Thus, we conducted a prospective longitudinal study among hematological cancer patients.

Studying post-traumatic growth in the case of cancer implies taking into account the specific circumstances related to this context. Sumalla et al. [17] indicate that cancer is a particularly aversive event and a number of characteristics need to be delineated. Among these, it may be difficult to identify a single stressor. There may be multiple stressors, including the diagnosis of the disease, its severity, the prognosis, the aggressiveness of treatment, etc. To limit confounding factors, we have only focused on HSCT in this study, and the participants were asked to give their feelings and thoughts only in reference to this aversive event. Thus, we conducted a prospective longitudinal study in which the primary purpose aimed to compare two methods of measuring post-traumatic growth using the same methodology as in Frazier et al.’s study (2009): a prospective one for actual growth and a retrospective one for perceived growth in the specific context of HSCT, a population under-explored about this issue. The secondary purpose aimed to put forward that both measures reveal different patterns of outcomes and distinct psychological correlates (mental health factors and psychological dispositions). Finally, to go further, we tested the hypothesis that acquiring positive outcomes from a traumatic event, such as HSCT, requires direct confrontation with the source of distress.

To compare retrospective and prospective measures of PTG, we drew inspiration from Frazier et al. [11]. Retrospective perception of growth was assessed with the PTGI at 5 months after HSCT. Prospective measure of growth was realised using scales that capture the five domains of growth assessed by the PTGI (i.e. relating to others, new possibilities, personal strength, spirituality change, and appreciation of life). Prospective measure of growth was done twice: during the week of the transplantation at the hospital and at the 5-month follow-up. We also examined the correlations between the five domains of retrospective and prospective measures of growth.

To go further in the study of the differences and similarities between both measures of PTG, we also studied their relationships with mental health and several psychological dispositions that we assessed 3 weeks prior to hospitalisation for HSCT. Concerning mental health, we selected anxiety, depression, and happiness. Concerning psychological dispositions, we selected optimism, acceptance, extraversion, and the five facets of dispositional mindfulness. According to the meta-analysis of Shand et al. [18], psychological growth following HSCT should be positively related to optimism and negatively related to poor mental health. In addition, recent research reveals that dispositional mindfulness and acceptance, a core construct of acceptance and commitment therapy, are beneficial psychological resources that could facilitate post-HSCT recovery [19, 20]. Thus, we predicted that acceptance and dispositional mindfulness, especially the non-judging and non-reacting facets [21], would facilitate genuine growth measured prospectively.

Finally, we tested the prediction that post-traumatic growth requires direct confrontation with the source of trauma using both retrospective and prospective measures. In their meta-analysis, Shand et al. [18] found a small and positive relationship between PTSD and post-traumatic growth in cancer patients (i.e. *r* = 0.13). We followed the rationale of Kashdan and Kane [22] in that unwillingness to be in contact with distressing thoughts and feelings—i.e. experiential avoidance—would moderate the relationship between Post-Traumatic Stress Disorder (PTSD) and growth. Thus, among patients who reported high experiential avoidance prior to HSCT, there should be no association between PTSD and psychological growth at the five-month follow-up. This association should appear only in patients with low experiential avoidance.

## Method

### Participants

**Fig. 1:**
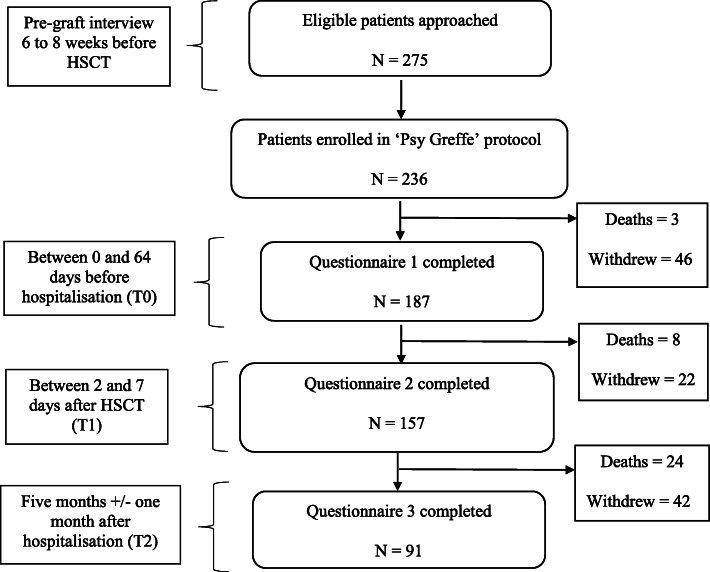
Flow diagram of protocol

The study protocol was presented to 275 patients. Of these, 236 signed the informed consent and entered in the ‘psygreffe’ cohort. Of these, 187 completed the first questionnaire (*M*_age_ = 52.07, *SD* = 13.22, ranging from 19 to 72 years old), 157 filled out the second questionnaire, and 91 filled out the third questionnaire.^1^ Between the completion of the first and the third questionnaire, 30 participants died. In addition, 67 participants left the study during the protocol for various reasons (e.g. fatigue, lack of motivation) (Fig. 1). Patients came from three hospital centers of Paris, Nancy, and Clermont-Ferrand in France. Forty-one- point 9 % of participants were female. In total, 65.7% were married, 46.3% had an educational level beyond the license degree, and 22.5% belonged to the upper-professional category. Seventeen percent had myelodysplastic syndrome, 10.4% had myeloproliferative neoplasia, and 35.7% were candidates for an allograft for acute leukemia. Ninety-four percent were having their first transplant (Table 1). In each hospital center, the patients interviewed could benefit from psychological support if needed. The ethical committee Sud-Est III (IRB 2017–026 B) approved the study. Informed written consent was obtained from each participant.

**Table 1:** Descriptive Statistics for Socio Demographic and Medical Variables at Time 0 and Time 2

	Time 0			Time 2		
	% (excluding missing values)	Mean (SD)	N	% (excluding missing values)	Mean (SD)	N
Controlled socio demographic variables						
Age		52.03 (13.28)	217		51.61 (12.93)	89
Sex *(women)*	42.7		221	42.7		89
Marital Status *(married)*	46.4		181	48		75
Educational Level *(post-graduate)*	46.3		175	46.1		76
Socio-professional Category *(employed)*	69.6		151		67.6	68
Follow-up (in months)		6.58 (4.04)				
Controlled medical variables						
Disease Status			178			80
*Acute Leukemia*	36			36.3		
*Myelodysplastic Syndrome*	17.4			13.8		
*Myeloproliferative Neoplasia*	10.1			8.8		
*Non-Hodgkin’s Lymphoma*	11.8			13.8		
Alcohol consumption *(yes)*	30.8		172	22.5		71
Smoking *(yes)*	15.8		177	8		75
Physical Activity *(yes)*	45.3		172	54.8		73
Body Mass Index		24.92 (4.61)	176		24.19 (4.22)	74
Sleeping hours		7.42 (1.15)	161		7.29 (1.15)	68
Number of transplantations		1.07 (0.3)	178		1.04 (.19)	80
Latency between disease		2.61 (4.41)	178		3.03 (4.69)	80
diagnostic and transplantation (*in years*)						
Myeloablative conditioning	25.8		178	25		80
Chronic GvHD	16.5		164	18.1		72
Donor type			179			80
*Identical sibling*	25.7			31.3		
*Mismatched unrelated*	8.9			8.8		
*Mismatched relative*	12.8			8.8		
*Matched unrelated*	38			41.3		
*Unrelated*	14			8.8		
*Matched other relative*	0.6			1.3		
Latency engraftment (*in days*)		20.24 (6.95)	161		19.85 (5.67)	73
Acute GvHD	51.5		171	57.9		76
Relapse	14.8		162	5.6		72
Number of infections		2.14 (1.8)	170		1.87 (1.82)	76
Death	16.4		177	1.3		80

### Procedure

All participants were informed of the study during the pre-graft interview (i.e. the doctor and medical staff give details about the allograft procedure, the expected benefits and related risks of the treatment, the functioning of the service, and so on) and read an information note. They had 15 days to decide whether they would participate or not. Then, they filled out an informed consent form and completed a first questionnaire assessing mental health (i.e. anxiety, depression, and happiness), psychological dispositions (i.e. optimism, extraversion, experiential avoidance, acceptance, and dispositional mindfulness) and sociodemographic variables (i.e. age, sex, marital status, and educational level) 20 days (*Mean* = 19.6, *SD* = 14.14) before their hospitalisation for an allograft (Time 0). A second questionnaire was given at the start of hospitalisation and had to be completed during the first week from the day after the allograft intervention (Time 1). This second questionnaire evaluated five dimensions of post-traumatic growth, which constitute the prospective measure of growth. Finally, participants were invited to complete a third questionnaire at 5 months after their entrance to the hospital during a follow-up date (Time 2). This third questionnaire measured the same five domains of growth that constitute the prospective measure of growth, a scale measuring growth retrospectively (PTGI), and a measure of Post-Traumatic Stress Disorder (PTSD). The relevant medical data were extracted from the ProMISe (Project Manager Internet Server).

### Measures

#### Post-traumatic growth (PTG) assessment

##### Retrospective measure of post-traumatic growth

To assess perceived change from pre- to post-trauma, participants were asked to complete the Post Traumatic Growth Inventory (PTGI) [23] 5 months after the HSCT with regard to the transplant they had experienced. This scale measures post-traumatic growth across 21 items. Individuals are asked to indicate on a scale ranging from 0 (‘I did not experience this change’) to 5 (‘I experienced this change significantly’) how much they experienced and lived with change in their life since HSCT. The total score is calculated by adding up each of the items. The internal consistency of the scale in our study is very satisfactory (*α* = 0.96). The PTGI measures five areas of growth: (1) relating to others (e.g. ‘I accept needing others’) (*α* = 0.89), (2) new possibilities (e.g. ‘I developed new interests’) (*α* = .90), (3) personal strength (e.g. ‘I discovered that I’m stronger than I thought I was’) (*α* = 0.86), (4) spirituality change (e.g. ‘I have a stronger religious faith’) (*α* = 0.78), and (5) appreciation of life (e.g. ‘My priorities about what is important in life’) (*α* = 0.85).

##### Prospective measure of post-traumatic growth on measures of PTG dimensions

To assess actual change from pre- to post-trauma, at both Time 1 and Time 2, we asked participants to complete several scales that corresponded to the dimensions of growth assessed by the PTGI. We examined whether the domain measures we chose assessed similar general constructs as the PTGI by correlating scores on the PTGI at Time 2 with scores on the five dimensions measured independently at Time 2. The first dimension, ‘relating to others’ was assessed using the 13-item Positive Orientation to Others dimension from the Goal and Mode Value Inventories [24] (e.g. ‘Accepting others even though they may be different from you’; *α*_t1_ *=* 0.92*; α*_t2_ *=* 0.93). The correlation with ‘relating to others’ (PTGI) was 0.41 (*p* < 0.001). The second dimension, namely ‘new possibilities’, was assessed with the Personal Growth subscale (14 items) from Ryff and Essex’s Psychological Well Being (PWB) scale [25] (e.g., ‘I think it is important to have new experiences that challenge how you think about yourself and the world’; *α*_t1_ *=* 0.79*; α*_t2_ *=* 0.79). The correlation between this measure and the new possibilities dimension of PTGI was 0.28 (*p* < 0.01). The third domain, ‘personal strength’, was measured using the 24-item Brief Strengths Test, which is a brief version of the ‘values in action inventory of strengths’ (VIAIS) [26] (e.g. ‘You are viewed as a creative person; you see, do, and/or create things that are of use; you think of unique ways to solve problems and be productive’). The 24-item scale had a satisfactory internal consistency (*α*_t1_ *=* 0.86*; α*_t2_ *=* 0.92) and correlated positively with the ‘personal strength’ dimension of the PTGI (*r* = 0.29, *p* < 0.01). The 24 personal strengths are grouped into six virtues (wisdom and knowledge, courage, humanity, justice, temperance, transcendence). The fourth dimension, ‘change in spirituality’, was assessed using the 6-item Intrinsic Spirituality scale of Hodge [27] (e.g. ‘In terms of the questions I have about life, my spirituality answers no questions / absolutely all my questions’; *α*_t1_ *=* 0.96*; α*_t2_ *=* 0.97). This scale correlated strongly with the ‘spirituality change’ dimension of the PTGI (*r* = 0.60, *p* < 0.001). Finally, ‘appreciation of life’, the fifth domain, was measured using the 5-item Satisfaction with Life Scale developed by Diener et al. [28] (e.g. ‘In most ways my life is close to my ideal’; *α*_t1_ *=* 0.91*; α*_t2_ *=* 0.85). Unexpectedly, and contrary to Frazier et al. [11], this scale was not related significantly to the appreciation of life component from the PTGI (*r* = 0.12, *p* > 0.25) A composite score of prospective measure of PTG was computed by averaging the five dimensions. At Time 2, this composite score was positively and significantly related to PTGI (*r* = 0.42, *p* < 0.001).

### Mental health and psychological disposition prior to hospitalisation

#### Mental health

We assessed anxiety, depression, and happiness. Anxiety and depression symptomatology was measured with the Hospital Anxiety and Depression scale (HADs) [29]. Seven items estimated anxiety symptomatology (*α*_t0_ = 0.76; *α*_t2_ *=* 0.72), and seven items assessed symptoms of depression (*α*_t0_ = 0.70; *α*_t2_ *=* 0.80). Happiness was assessed with the Subjective Authentic-Durable Happiness scale (SA-DHS) [30] (*α*_t0_ = 0.96; *α*_t2_ *=* 0.97).

#### Psychological dispositions

While optimism was measured using the Life Orientation Test- revised (LOT-R; *α* = 0.76) [31], extraversion was assessed using the Big Five Inventory (BFI; *α* = 0.84) [32]. Experiential avoidance was measured with the Avoidance and Fusion Questionnaire for Adults (AFQ**;** *α* = 0.88) [33], and acceptance was assessed with the Acceptance and Action Questionnaire II (AAQ II; *α* = 0.81) [34]. Dispositional mindfulness was assessed with the FFMQ [35] (*α* = 0.87). This scale comprises five dimensions: observing, describing, acting with awareness, nonjudging, and nonreactivity to the experience.

#### PTSD assessment at follow-up

The Post-Traumatic Stress Disorder Checklist Scale (PCLS) [36] was used to detect post-traumatic stress disorder through 17 items assessing the severity of 17 symptoms of PTSD listed in the DSM-V. This scale had an adequate internal consistency (*α* = 0.91) (For all measures references see Supplementary File).

## Results

### Retrospective and prospective measures of growth

Table 2 presents descriptive data. Concerning the prospective measure of growth, we compared the scores obtained at the five-month follow-up with the scores assessed before HSCT. For each comparison, we reported the *p*-value and BF_10_ (i.e. the extent to which the data support H_1_). Substantial support for H_1_ was provided by a BF > 3 (BF > 10 was judged strong; > 30 very strong and > 100 decisive). Support for H_0_ was provided by a BF < 1 [37].

**Table 2:** Descriptive Data of Retrospective and Prospective Measures of Post-Traumatic Growth

	Mean before HSCT	Mean at 5-month follow-up	Change score	BF_10_	Reliable increase	Reliable decrease
*Retrospective Measure of Growth (PTGI)*	–		–	–	–	–
- Relating to others	–	2.90	–	–	–	–
- New Possibilities	–	2.30	–	–	–	–
- Personal strength	–	2.84	–	–	–	–
- Change in spirituality	–	1.84	–	–	–	–
- Appreciation of life	–	3.23	–	–	–	–
Mean score of PTGI	–	2.67	–	–	–	–
*Prospective Measure of Growth*						
- Positive orientation to others	5.81	5.35	−0.46***	65	8.5%	33.8%
- Personal Growth subscale	4.67	4.53	−0.14*	0.9	11.4%	28.6%
- Brief Strengths Inventory	3.86	3.70	−0.16**	7.8	10.0%	28.6%
Wisdom/knowledge	3.87	3.70	−0.17**	3.3	7.1%	25.7%
Courage	3.85	3.79	−0.06	0.2	10.3%	10.3%
Humanity	4.03	3.84	−0.19*	2.5	10.3%	27.9%
Justice	3.96	3.62	− 0.34***	31.9	2.9%	16.2%
Temperance	3.59	3.48	−0.11	0.2	8.8%	10.3%
Transcendence	3.92	3.76	−0.16*	0.9	8.8%	17.6%
- Intrinsic Spirituality scale	4.80	4.16	−0.64**	6.7	17.4%	43.5%
- Life satisfaction scale	5.40	4.92	−0.48***	52.8	8.6%	32.9%
Mean score of growth	4.90	4.52	−0.38***	> 150	5.6%	40.8%

*** *p* < 0.001, ** *p* < 0.01, * *p* < 0.05

Four of the five dimensions we measured showed a significant decrease between before and after transplantation (i.e. positive orientation, personal strengths, spirituality, and life satisfaction). Bayesian factors also provided clear support for the hypothesis of a decrease following HSCT for these four measures. The only dimension that did not vary significantly with time was the personal growth subscale from the psychological well-being scale [25]. The reliable change index (RCI) was computed for each dimension [38]. The percentage of reliable decrease was always superior to the percentage of reliable increase. The RCI for the mean score of change in growth indicated a reliable increase for 5.6% of patients and a reliable decrease for 40.8% of patients.

### Relationship between retrospective and prospective measures of growth

The mean score of change in growth (actual growth at Time 2 – actual growth at Time 1) was significantly and weakly related to the mean score of PTGI assessed at Time 2 (*r* = 0.25, *p* < 0.036). As Table 3 shows, of the five dimensions in retrospective and prospective measures of growth assessed, two dimensions were significantly correlated (i.e. personal strengths and appreciation/satisfaction with life) and three domains were not significantly correlated (i.e. relating/positive orientation to others, new possibilities/personal growth subscale, and spirituality).

**Table 3:** Correlations Between Retrospective Measure of Growth (PTGI) and Change in Growth (T2-T1)

	Change in Growth (T2 – T1)				
	Positive orientation to others	Personal Growth subscale	Brief Strengths Inventory	Intrinsic Spirituality scale	Life satisfaction scale
*Retrospective Measure of Growth at Time 2 (PTGI)*					
Relating to Others	**0.17**	0.12	0.29*	0.20+	0.10
New Possibilities	0.15	**0.16**	0.29*	0.03	0.17
Personal Strength	0.15	0.03	0.29*	0.07	0.17
Spiritual Change	0.12	0.26*	0.03	**− 0.01**	0.09
Appreciation of life	0.19	0.04	0.29*	−0.05	0.25*
*Mean score of PTGI*	0.18	0.13	0.29*	0.08	0.18

* *p* < 0.05, + *p* < 0.10

### Relationship between retrospective and prospective measures of growth, and mental health and psychological dispositions

Table 4 presents the correlations between the measures of growth and those of mental health and psychological dispositions. Concerning mental health, while happiness prior to hospitalisation significantly predicted growth measured retrospectively (*r* = 0.36, *p* < 0.001), change in growth measured prospectively was not significantly related to happiness (*r* = 0.14). Thus, the happiest patients prior to hospitalisation were those who perceived greater growth at the follow-up.

**Table 4:** Prospective Effects of Mental Health and Positive Psychological Disposition Prior to Hospitalization for HSCT (Time 0) on Retrospective Measure of Growth (PTGI) at Time 2, and Change in Growth Between Time 1 and Time 2

	Anxiety (HAD-A)	Depression (HAD-D)	Happiness (SA-DHS)	Optimism (LOT)	Extraversion (BFI)	Acceptance (AAQII)
*Retrospective Measure of Growth at Time 2 (PTGI)*						
Relating to Others	−0.05	− 0.11	0.42***	0.30**	0.22+	0.15
New Possibilities	0.17	0.09	0.23*†	−0.01	0.18	−0.06
Personal Strength	−0.06	−0.10	0.35***	0.13	0.23*	0.10†
Spiritual Change	−0.01	−0.07	0.23*†	0.13	0.19†	− 0.02
Appreciation of life	0.05	−0.06	0.26*	0.15	0.32**	0.09
Mean score of PTGI	0.03	−0.05	0.36***	0.17	0.25*	0.06†
*Change in Growth (Time 2 – Time 1)*						
Positive orientation to others	−0.17	− 0.29*	0.21	0.03	0.24+	0.22+
Personal Growth subscale	0.38**	0.20	−0.23+	−0.28*	− 0.06	− 0.09
Brief Strengths Inventory	− 0.13	−0.20	0.16	0.20	0.26*	0.50*
Intrinsic Spirituality scale	−0.07	−0.20	0.11	0.15	−0.18	0.24+
Life satisfaction scale	−0.19	0.03	0.02	0.02	0.07	0.21
Mean score in growth change	−0.14	−0.20	0.14	0.11	0.04	0.39**

*** *p* < 0.001, ** *p* < 0.01, * *p* < 0.05, + *p* < 0.10; A correlation with the retrospective measure of growth accompanied by † differs to 0.05 with the corresponding correlation with prospective measure of growth

Extraversion prior to hospitalisation (*r* = 0.25, *p* < 0.05), but not optimism and acceptance (respectively, *r* = 0.17 and *r* = 0.06, *p*s > 0.10), was found to significantly and positively predict the PTGI. This was not the case with change in growth, measured prospectively, which was positively and significantly related only to acceptance (*r* = 0.39, *p* < 0.01). Thus, the most extraverted patients prior to hospitalisation were those who reported the highest level of PTGI at the follow-up, and those who scored higher on the acceptance scale prior to hospitalisation benefited the most in terms of growth between transplantation and the five-month follow-up. Concerning dispositional mindfulness (see Table 5), observing and describing were significantly and positively related to the retrospective measure of growth (respectively, *r* = 0.32 and *r* = 0.34, *p*s < 0.01), but not to the prospective measure of growth (respectively, *r* = 0.16 and *r* = 0.23, *p*s > 0.05). The nonjudgment facet was negatively and significantly related to the retrospective measure of growth (*r* = − 0.24, *p* < 0.05) and positively and significantly related to change in growth (*r* = 0.28, *p* < 0.05). The latter was positively and marginally related to non-reacting (*r* = 0.24, *p* < 0.06), which was not the case with the retrospective measure of growth (*r* = 0.06).

**Table 5:** Prospective Effects of Five Facets of Mindfulness Prior to Hospitalisation for HSCT (Time 0) on Retrospective Measure of Growth (PTGI) at Time 2 and Change in Growth Between Time 1 and Time 2

	Five Facets Mindfulness Questionnaire (FFMQ)				
	Observing	Describing	Acting with awareness	Non-reacting	Non-judging
*Retrospective Measure of Growth at Time 2 (PTGI)*					
Relating to Others	0.30**	0.33**	0.13	0.16	−0.11
New Possibilities	0.28*	0.27*	0.08	−0.04	− 0.26*†
Personal Strength	0.24*	0.28*	0.21+	0.02	−0.17†
Spiritual Change	0.30**†	0.22+	0.05	0.08	−0.21 + †
Appreciation of Life	0.30**	0.37***	0.26*	0.01	−0.29*
Total Score	0.32**	0.34**	0.16	0.06	−0.24*†
*Change in Growth (Time 2 – Time 1)*					
Positive orientation to others	0.21	0.19	0.22	0.26*	0.09
Personal Growth subscale	0.05	0.11	−0.15	0.04	0.14
Brief Strengths Inventory	0.31*	0.29*	0.19	0.18	0.23+
Intrinsic Spirituality scale	−0.06	0.01	0.14	0.17	0.24+
Life satisfaction scale	0.15	0.17	0.12	−0.03	0.05
Mean score in growth change	0.16	0.23+	0.23+	0.24+	0.28*

*** *p* < 0.001, ** *p* < 0.01, * *p* < 0.05, + *p* < 0.10; A correlation with the retrospective measure of growth accompanied by † differs by 0.05 with the corresponding correlation with prospective measure of growth

### Test of the moderating effect of experiential avoidance on the relationship between post-traumatic stress disorder (PTSD) and the retrospective/prospective measure of growth

We centred all the variables on the grand mean. Using Process Version 3.4.1. for SPSS, we performed a moderation analysis (Bootstrap: 5000 samples) [39] with PTSD as the independent variable, experiential avoidance as a moderator, and the retrospective measure of growth as a dependent variable (DV). While PTSD was marginally and positively related with growth when measured retrospectively (b = 0.02, SE = 0.01, *p* < 0.06), experiential avoidance was not related to PTGI (b = 0.14, SE = 0.22, *p* > 0.50). We found support for a PTSD x experiential avoidance interaction effect in predicting post-traumatic growth measured retrospectively (PTGI; b = − 0.03, SE = 0.01, *p* < 0.038). Conditioned at 1 SD below the mean for experiential avoidance, PTSD was positively related to PTGI (b = 0.04, SE = 0.02, *p* < 0.02), whereas when conditioned at 1 SD above the mean for experiential avoidance, PTSD was not related to PTGI (b = 0.01, SE = 0.01, *p* > 0.70). Thus, in the absence of experiential avoidance, PTSD was associated with greater perceived growth as assessed by PTGI. This was not the case in the presence of experiential avoidance. Finally, we computed a similar moderation analysis with the mean score of change in growth measured prospectively as a DV. Neither PTSD nor experiential avoidance were related to change in growth (all *p*s > 0.10). We did not find support for a PTSD x experiential avoidance interaction effect (b = − 0.01, SE = 0.01, *p* > 0.45).

## Discussion

Focusing on post-traumatic growth among patients who have received an allograft, the primary aim of this study was to compare two methods of measuring post-traumatic growth: a prospective one for actual growth and a retrospective one for perceived growth in the case of HSCT. To address this primary aim, we compared scores on perceived post-traumatic growth, assessed by the Post-Traumatic Growth Inventory (PTGI) [8], and changes in scores of post-traumatic growth measured at two times (during the first week after transplantation and 5 months later) and corresponding to the five domains of PTGI. With a secondary objective, this research also aimed to highlight the different characteristics of retrospective and prospective measures of growth with respective relationships between these two measures of growth and both mental health and psychological dispositions, with a special investigation on dispositional mindfulness facets and experiential avoidance as a moderator between PTSD and both measures of growth.

Firstly, results seem to more support the hypothesis of decline rather than growth in the case of a prospective measure of post-traumatic growth 5 months after HSCT. These findings are consistent with the study of Frazier et al. [11], who found that post-traumatic growth, when assessed with a pre/post-test methodology is not as common as previously shown in numerous studies and can be a skewed perception of positive changes. More specifically, assessment instruments of post-traumatic growth, such as the PTGI—the most commonly used instrument to assess growth following an adverse event [14]—are not suitable for revealing a genuine decline or increase of growth. This suggests there is a need to develop more reliable instruments of post-traumatic growth [40] and conduct more systematic, longitudinal protocols, as recommended by Infurna and Jayawickreme [12]. In addition, the results of this study clearly raise questions about the specificities of the allograft process. Indeed, as highlighted by Sumalla et al. [17], research on post-traumatic growth should consider the substantial differences existing between the traumatic nature of cancer and an acute trauma, leading consequently to different health outcomes. For example, contrary to an acute stress, the temporal delimitation of the traumatic event in the case of cancer is not as perceptible or delimited for various reasons such as the risk of relapse. These differences have major implications on the lived experiences of patients who have to face an ongoing threat, which often engenders psychological distress. This observation is particularly true in the case of HSCT since patients are constantly confronted with the risk of relapse, infections, and graft versus host disease (GvHD) in the long-term, despite an improvement of patients’ quality of life over time revealed by some studies. So, a decline in psychological growth could reflect the specificities of such treatments. It would be relevant to conduct a follow-up several years after HSCT to determine whether patients present a complete or a partial psychological recovery [41].

Other findings aimed at answering our secondary objective as revealed by this study include the weak correlation between retrospective and prospective measures of growth and the fact that they have different psychological correlates [9, 11]. If growth assessed by PTGI is mainly related to positive thoughts and feelings such as happiness [18] and a personality trait (i.e. extraversion [23];), then growth measured prospectively is, however, only predicted by a characteristic of psychological flexibility, namely acceptance. The nature of psychological correlates of the retrospective measure of growth tends to support the interpretation of Infurna and Jayawickreme [12] who suggest that PTGI may reflect reality distortions, selective appraisals, coping, and personality characteristics. However, the prospective effect of acceptance on change in growth suggests the need to target preventively this dimension of psychological flexibility to alleviate psychological distress and enhance actual psychological growth among patients confronted with stressful events such as cancer [20] and HSCT.

Concerning dispositional mindfulness, with the exception of non-judging, most facets (i.e. observing, describing, acting with awareness, non-judging) did not robustly predict change in growth. This is not the case for the retrospective measure of growth, which is positively predicted by the observing and describing facets, whereas the non-judging dimension is negatively related to this measure of growth. These results seem to confirm that growth, when assessed retrospectively, is linked to a cognitive activity of interpreting lived experience that involves observing, describing, and judging one’s inner experience, while change in growth is more related to decentering (here to non-judging) and acceptance processes.

The assumption made about the moderating effect of experiential avoidance on the relationship between PTSD and both measures of growth confirms the study of Kashdan and Kane [22] for retrospective measure of growth only. For the prospective measure of growth, this confirms the meta-analysis of Mangelsdorf et al. [13], who concluded that there is ‘no general evidence for the widespread conviction that negative life events have a stronger effect than positive ones. Therefore, a direct confrontation with the source of distress does not allow for actual growth: it is rather its acceptance and lack of over-judging that seems important. On the other hand, as for Kashdan and Kane [22], this direct confrontation seems to be associated with retrospective perception of growth. This may once again depict the interpretive activity of lived experience. Those who are in avoidance do not interpret and do not positively re-evaluate their experiences. Consistently, research has revealed that avoidance coping is negatively related with positive re-appraisal coping [42], the latter being involved in the perception of a post-traumatic growth [11].

## Conclusions

To conclude the main results extracted from this study, we can observe, in the case of HSCT, a decline at 5 months when post-traumatic growth is measured prospectively, which suggests that a substantial portion of patients encounter impairments in their psychological state even a few months after the intervention. Therefore, it seems particularly relevant to identify post-HSTC difficulties that inhibit growth in order to remedy them. For example, future studies may examine the role of factors such as the consequences of transplantation (e.g. high risk of complications, physical and psychological sequelae) during the following months and years after transplantation [4, 5] or socio-demographic factors such age, which can influence the lived experience of post-traumatic growth. Despite the need for further studies with a more long-term follow-up and more suitable scales tapping into the five domains of the Post-Traumatic Growth Inventory to characterise the nature of changes lived by patients, the prospective effect of acceptance and non-judging on actual growth offers an interesting perspective for prevention.

## Supplementary Information

**Additional file 1.** Measures References

## Data Availability

Datasets of this study are available on 10.6084/m9.figshare.12382916.v2

## Abbreviations

HSCT: Hematopoietic Stem Cell Transplantation
PTSD: Post-Traumatic Stress Disorder
PTG: Post-Traumatic Growth
PTGI: Post-Traumatic Growth Inventory
RCI: Reliable Change Index
PWB: Psychological Well-Being
VIAIS: Values in Action Inventory of Strengths
HADs: Hospital Anxiety and Depression scale
SA-DHS: Subjective Authentic-Durable Happiness scale
LOT-R: Life Orientation Test-Revised
BFI: Big Five Inventory
AFQ: Avoidance and Fusion Questionnaire
AAQ: Acceptance and Action Questionnaire
FFMQ: Five Facets Mindfulness Questionnaire
PCLS: Post-Traumatic Stress Disorder Checklist Scale
BF: Bayesian Factor
DV: Dependant Variable
SD: Standard Deviation
GVHD: Graft Versus Host Disease

## Footnotes

1 We estimated the required sample size for sufficient statistical power using G*Power 3.1 (power = 80%). First, concerning the comparison in prospective growth between time 1 and time 2, we selected ‘Anova: Repeated measures, within factors’ (Number of groups = 1; Number of measurements = 2; correlation among repeated measures = 0.5) with a small effect size (i.e. Partial eta-squared = 0.05, Effect size f = 0.229). The minimum required sample size was 52. Second, on the basis of the correlation between coping and PTGI reported by Frazier et al. (2009; i.e. *r* = 0.52, *r*^2^ = 0.27), the minimum required sample size was 26. Finally, concerning the moderation hypothesis predicted based on Kashdan & Kane (2011), we selected ‘Linear multiple regression: Fixed model, R^2^ deviation from zero’ (Number of predictors = 3). Then, we specified predictor correlations using beta coefficients between predictors and outcome provided by Kashdan & Kane in their Table 2 (2011; i.e. 0.04 for PTSD, 0.08 for experiential avoidance, and − 0.35 for the interaction between PTSD and experiential avoidance). The obtained squared multiple correlation was 0.1293 and the effect size f^2^ was 0.1485. The minimum required sample size was 78.
