# Supplementary material for: Retrospective and prospective measures of post-traumatic growth reflect different processes: longitudinal evidence of greater decline than growth following a hematopoietic stem-cell transplantation
Source: BMC Psychiatry. 2021 Jan 11;21:27. doi: 10.1186/s12888-020-03007-y (PMC7798346; doi:10.1186/s12888-020-03007-y)
Supplement: Supplementary file 1 — Additional file 1. Measures References [file 12888_2020_3007_MOESM1_ESM.docx]

Measures References

Note : all references are mentioned in manuscript. When full version of English version of scale aren’t available in papers, a link toward it is added.

**Post Traumatic Growth Inventory (PTGI)**

Tedeschi RG, Calhoun LG. The Posttraumatic Growth Inventory: Measuring the positive legacy of trauma. Journal of traumatic stress. 1996; 9(3): 455-471.

# Positive Orientation to Others dimension from the Goal and Mode Value Inventories

Braithwaite VA, Law HG. Structure of human values: Testing the adequacy of the Rokeach Value Survey. Journal of personality and social psychology. 1985; 49(1): 250.

# Personal Growth subscale from Ryff and Essex’s Psychological Well Being (PWB) scale

Ryff CD, Essex MJ. The interpretation of life experience and well-being: The sample case of relocation. Psychology and aging. 1992; 7(4): 507.

<https://docs.google.com/document/d/1pn2oZi3NiSuEJjtxl0hfaFpEa4Ea0v6YtrlWTAkVUlY/edit>

**24-item Brief Strengths Test, a brief version of the ‘values in action inventory of strengths’ (VIAIS)**

McGrath, R. E. (2017). Technical report: The VIA Assessment Suite for Adults: Development and evaluation. Cincinnati, OH: VIA Institute on Character.

<https://www.viacharacter.org/pdf/Technical%20Report%20Revised%20Edition%202019_1.pdf>

# Intrinsic Spirituality scale

Hodge DR. The intrinsic spirituality scale: A new six-item instrument for assessing the salience of spirituality as a motivational construct. Journal of Social Service Research. 2003; 30(1): 41-61.

# Satisfaction with Life Scale

Diener ED, Emmons RA, Larsen RJ, Griffin S. The satisfaction with life scale. Journal of personality assessment. 1985 ; 49(1): 71-75.

**Hospital Anxiety and Depression scale (HADs)**

Zigmond AS, Snaith RP.The hospital anxiety and depression scale. Acta psychiatrica scandinavica. 1983; 67(6): 361-370.

**Subjective Authentic-Durable Happiness scale (SA-DHS)**

Dambrun M, Desprès G, Lac G. Measuring happiness: from fluctuating happiness to authentic–durable happiness. Frontiers in psychology. 2012 ; 3 : 16.

# Life Orientation Test- revised

Scheier MF, Carver CS, Bridges MW. Distinguishing optimism from neuroticism (and trait anxiety, self-mastery, and self-esteem): a reevaluation of the Life Orientation Test. Journal of personality and social psychology. 1994; 67(6) : 1063.

# Big Five Inventory

John OP, Srivastava S. The Big Five trait taxonomy: History, measurement, and theoretical perspectives. Handbook of personality: Theory and research. 1999 ; 2 : 102-138.

# Avoidance and Fusion Questionnaire for Adults

Corman M, Dambrun M, Bay JO, & de La Tour RP. Adaptation française et analyse des qualités psychométriques du questionnaire d’évitement et de fusion (AFQ) dans une population adulte. In Annales Médico-psychologiques, revue psychiatrique. 2019; 177(4): [358-363](tel:358363).

# Acceptance and Action Questionnaire II

Bond FW, Hayes SC, Baer RA, Carpenter KM, Guenole N, Orcutt HK, et al. Preliminary psychometric properties of the Acceptance and Action Questionnaire–II: A revised measure of psychological inflexibility and experiential avoidance. Behavior therapy. 2011; 42(4): 676-688.

# Five Facets Mindfulness Questionnaire

Baer RA, Smith GT, Hopkins J, Krietemeyer J, Toney L. Using self-report assessment methods to explore facets of mindfulness. Assessment. 2006; 13(1): 27-45.

**Post-Traumatic Stress Disorder Checklist Scale**

Weathers FW, Litz BT, Herman DS, Huska JA, Keane TM. The PTSD Checklist (PCL): Reliability, validity, and diagnostic utility. In annual convention of the international society for traumatic stress studies, San Antonio, TX; 1993.
